# Multisensory stimulation bundles on sleep and neurobehavioral development in the first year after birth in very preterm infants: a randomized crossover controlled study protocol

**DOI:** 10.1186/s13063-023-07753-8

**Published:** 2023-11-15

**Authors:** Xiaoli Tang, Sha Sha, Nanping Shen, Zhiyu Zhu, Yanmin Qin, Junyi Shen, Fei Bei

**Affiliations:** 1 Department of Neonatology, Shanghai Children’s Medical Center Affiliated to Shanghai Jiaotong University School of Medicine, National Children’s Medical Center (Shanghai), Shanghai, China; 2 Department of Nursing Department, Shanghai Children’s Medical Center Affiliated to Shanghai Jiaotong University School of Medicine, National Children’s Medical Center (Shanghai), Shanghai, China

**Keywords:** Multisensory, NICU, Preterm infant, Sensory stimulations

## Abstract

**Background:**

Disrupted sleep is believed to contribute to short- and long-term neurodevelopmental problems in very preterm infants (VPIs). This study presents a protocol for an evaluator-blinded, randomized crossover trial. It aims to assess the sleep efficiency of hospitalized VPIs by providing multisensory stimulation bundles. Furthermore, it aims to observe the intervention impacts on sleep during hospitalization of the VPIs and their sleep and neurodevelopmental outcomes during the first year of post-discharge follow-up.

**Methods:**

The study will be conducted in the neonatology department of a tertiary pediatric teaching hospital. All the eligible VPIs will undergo two types of care in random order: “standard care” (2 weeks) and “standard care plus multisensory stimulation bundles,” each lasting 2 weeks. A generated list of random numbers will be used for case sequence allocation. Sleep outcomes will be evaluated using the Actiwatch-2 Actigraph. Moreover, the amplitude-integrated electroencephalography and the Griffiths Mental Development Scales will be used to measure the neurodevelopmental outcomes during hospitalization and in the first year of follow-up of the VPIs.

**Discussion:**

The intervention protocol of this study differs from that of other traditional interventions by producing precise and consistent supportive stimulations, similar to maternal tactile, auditory, posture, and visual effects for hospitalized VPIs. This protocol could be an effective measure to facilitate sleep and early neurodevelopment of VPIs. The expected outcomes will help confirm the implementation and generalization of the multisensory stimulation bundles’ care protocol in neonatology departments. We expect that the study will positively impact hospitalized VPIs, especially in their sleep and early neurodevelopmental outcomes. It will also provide a new perspective regarding parent and infant interaction strategies, particularly for newborn intensive care units that limit visits because of the global spread of COVID-19.

**Trial registration:**

Chinese Clinical Trial Registry ChiCTR 2200059099. Registered on 25 April 2022, https://www.chictr.org.cn/showproj.html?proj=166980; the Hospital Research Ethics Committee (approval number: SCMCIRB-K2021086-1, Version 01), approved on 21 January 2022.

**Supplementary Information:**

The online version contains supplementary material available at 10.1186/s13063-023-07753-8.

## Introduction

### Background and rationale

More than 15 million preterm infants are born worldwide every year, and China accounts for over 1.8 million cases [1]. The World Health Organization defines “preterm infants” as those born before 37 weeks of gestation. Infants delivered between a gestational age (GA) of ≥28 weeks and <32 weeks are classified as “very preterm” [2]. Despite a significant increase in the treatment rate of very preterm infants (VPIs), 25% are noted to have behavioral impairments characterized by inattention to environmental simulation, low tolerance for stress, fussiness, irritability, and specific socio-emotional problems [3, 4]. Moreover, studies have shown that about 40–50% of VPIs experience varying degrees of neurodevelopmental impairments throughout their childhood [1, 4].

Sleep is a primary brain activity during the late fetal and early neonatal stages of human development. It is a process that enables neuronal restitution at the behavioral level and detoxification at the cellular level, for all humans particularly in preterm infants [5]. It is the foundation for developing physiological maturation, arousal regulation, and cognitive growth [5]. According to epidemiological studies, sleep deprivation or disturbed sleep, along with significant morbidity in neural development, triggers a variety of somatic and psychosocial disorders [5, 6].

### Sleep mechanisms in the neural development of very preterm infants

Safeguarding sleep and its cycles is crucial for the growth and early neurosensory development of preterm infants in neonatal intensive care units (NICUs). Their sleep patterns include active sleep (AS), quiet sleep (QS), and indeterminate sleep (IS); QS and AS can be identified from as early as 32 weeks of gestation [7]. It is recognized that AS is essential for the structural maturation of the central nervous system. Interestingly, QS promotes the restoration of energy reserves and cellular repair, and IS is characterized as the transitional stage between these two types of sleep patterns [7].

Prior studies have found that rapid eye movement (REM) time, also included in AS in preterm infants, has been well-documented for the maturation of fetal and neonatal brains in animal models [7, 8]. Sensory feedback from myoclonic twitches during REM sleep can trigger central neural oscillations, promoting neurodevelopmental processes (e.g., synapse formation, neuronal differentiation, and migration), and permits functional connectivity in developing brain networks [8]. Moreover, Arzoumanian et al. [8] indicate that a lack of REM sleep in early brain development might lead to neurobehavioral problems, sleep disturbances, and even reduced cerebral cortical size.

### Sensory-related sleep risks for VPIs during NICU hospitalization

Sleep undergoes progressive changes during intrauterine and extrauterine life in preterm infants. These changes depend heavily on GA [5, 9]. After preterm birth, infants are exposed to the NICU environment, which includes various sources of stress like excessive noises, prolonged high-intensity lighting, and frequent invasive procedures for neonates [10]. Moreover, hospitalized VPIs usually face long periods substantially lacking organized, cyclic, and multimodal stimulation across the senses, which, in general, the human fetus experiences in the uterus [9–12]. Hence, it can be deduced that sensory exposure and abatement will contribute to sleep deprivation and disorder risk in VPIs during NICU hospitalization.

### Effects of sensory-based interventions on VPIs during NICU hospitalization

Several stress stimuli cause sleep deprivation or disorders during NICU hospitalization. Fortunately, several developmental care measures have been thoroughly developed and are widely used in clinical settings to minimize the adverse stimuli. These developmental care interventions include setting patients in single rooms or arranging care or treatment procedures, such as routine lab blood drawing, x-rays, or physical assessments, according to the infant’s sleep cycle, to promote the length of AS time [5, 13]. Contrastingly, many existing studies address the effective sensory abatement for preterm infants in NICUs. Therefore, it can be deduced from earlier findings that sensory-based interventions are aimed at modifying the deprived environment. This approach simultaneously provides sensory-based stimulation similar to the intrauterine environment through individual or multimodal methods and synchronized sensory stimulations [9, 10, 14]. Thus far, most sensory-based interventions could be categorized as visual, vocal, and tactile stimulation and postural/motor support using remolded mattresses [9, 14].

### Single sensory-based stimulations

In previous studies, NICU sensory-based stimulation has positively affected immediate physiological stability and sleep patterns; however, the impacts on lifelong developmental progression are still under debate [14]. Recent systematic reviews have demonstrated that maternal heartbeat and lullabies had beneficial effects on preterm infants’ stability in both physiological and behavioral domains [15, 16]. Moreover, a meta-analysis revealed that total sleep time efficiency and active sleep efficiency were significantly greater in participant groups that had mattress remolding than control groups [17]. This highly recommended approach works primarily because mattress remolding allows infants to assume a position similar to that in the uterus [17]. Furthermore, another meta-analysis demonstrated that cycled light, as opposed to dim or irregular lighting, could facilitate a smooth transition for infants from the intrauterine to the extrauterine environment [18]. This light control intervention impacts the rhythmic production of various stress response hormones, improves respiratory and cardiac functions, supports sleep-wake state maturation, and enhances alertness in VPIs [18].

### Multisensory stimulation

The *Enriched Environment* and *Synactive Model of Infant Development* theories suggest that multimodal sensory stimulation has better neurological effects than a single stimulus [19–21]. Multimodal sensory stimulation can produce inter-sensory redundancy, enhancing fetal perception and information processing [20]. A well-known example is the Kangaroo Care method, which corroborates the positive effects of multisensory stimulation on preterm infants [22]. This redundancy occurs when the mother carries her child in her arms while singing and gently touching or rhythmically rocking the child and making eye contact [20, 22]. Kitase [23] found that preterm infants increased sleep time after receiving a new swaddling cloth, which provides touch and movement support. Furthermore, two studies have shown that the SENSE multisensory program had a positive effect on the neurobehavioral performance of VPIs at term-equivalent age [11, 24].

Notably, most multisensory stimulations have aimed to provide a setting similar to the intrauterine environment, which is why parents are the first choice to provide interventions for their infants [14]. However, since VPIs typically stay in the incubator, it could be challenging or impossible for their parents to continuously conduct Kangaroo Care or a SENSE program [25]. Additionally, some literature has reported a negative impact of maternal stress and postpartum depression on the quality of early maternal-infant interactions, especially for VPIs [25, 26]. With the spread of COVID-19 worldwide, most NICUs in China have restricted parental contact [26]. Thus, developing an effective method other than Kangaroo Care or parental sensory-based care for VPIs is essential. Few studies have reported on multisensory stimulation, especially for VPIs. Hence, exploring the effect of a well-designed environment, similar to the uterus, for preterm infants using a device that could provide multimodal sensory stimulations is worthwhile.

### Objectives

This study aims to analyze the efficacy of multisensory stimulation bundles for VPIs during hospitalization to improve their sleep, early neurodevelopment, and other associated outcomes (i.e., weight, length, and head circumference). The specific objectives of the study are as follows:

- To assess the effect of the multisensory stimulation bundles on sleep efficiency in VPIs during hospitalization.
- To observe the impact of sleep during hospitalization on the sleep and neurodevelopmental outcomes in the first-year post-discharge follow-up.

## Methods/design

### Study design and setting

This study was designed as a prospective, evaluator-blinded, randomized crossover trial, in accordance with the Standard Protocol Items: Recommendations for Interventional Trials guidelines. This study is conducted in the neonatology department of Shanghai Children’s Medical Center, a pediatric tertiary teaching hospital, and the National Children’s Medical Center in China. The study protocol has been approved by the Hospital Research Ethics Committee (approval number: SCMCIRB-K2021086-1) and is registered in the Chinese Clinical Trial Registry (Registry Number: ChiCTR2200059099).

### Participants

This study proposes the following inclusion criteria: (1) infants who are clinically stable in a NICU, born at a minimum of 28 weeks and less than 32 weeks GA; (2) birth weight between the 10th and 90th percentiles of the average birth weight for the same GA, identified as appropriate [2]; and (3) parents agreeing to participate in the study and for follow-up diagnosis and treatment at the designated hospital for one year after discharge. The exclusion criteria will include the following: (1) congenital malformation in the infant that interferes with the intervention or for which the intervention interferes with the required care (e.g., complex congenital heart disease, complex gastrointestinal malformations, diaphragmatic hernia); (2) severe nervous system malformations or disorders (e.g., bilirubin encephalopathy, central nervous system infection, intracranial hemorrhage of ≥degree III); (3) independent predictors of disturbed sleep or neurodevelopment (e.g., inborn metabolism errors, preeclampsia, bronchopulmonary dysplasia); and (4) visual or hearing impairment. Those with the following criteria would be exited from the study: (1) terminating treatment or death during the study period or (2) adverse clinical events likely associated with receiving the multisensory stimulation bundles, or severe adverse events possibly related to the multisensory stimulation bundles.

### Blinding

The nature of the intervention prevents blinding of outcome collectors because they can observe whether the baby receives “multisensory simulation bundles” in the NICU or not. Hence, an independent outcome-evaluation team that is not involved in the implementation of the study will be invited to assess the outcomes. This team will comprise an investigator, a physician, and two research assistants. The investigator and the physician will be responsible for interpreting the actigraphy and amplitude-integrated electroencephalography (aEEG) data, respectively. The two research assistants, with international Griffiths Mental Development Scales (GMDS) qualifications, will perform Griffiths assessments and provide evaluations. Furthermore, data analysts will be blinded to the participant allocation as much as possible.

### Randomization and allocation

The study participants will be randomly assigned to group A or B with a distribution ratio of 1:1. A statistician has prepared a computer-generated randomization list with variable block sizes. The random allocation will be concealed in serially numbered, opaque, and sealed envelopes prepared for the site.

### Screening and enrollment

**Fig. 1:**
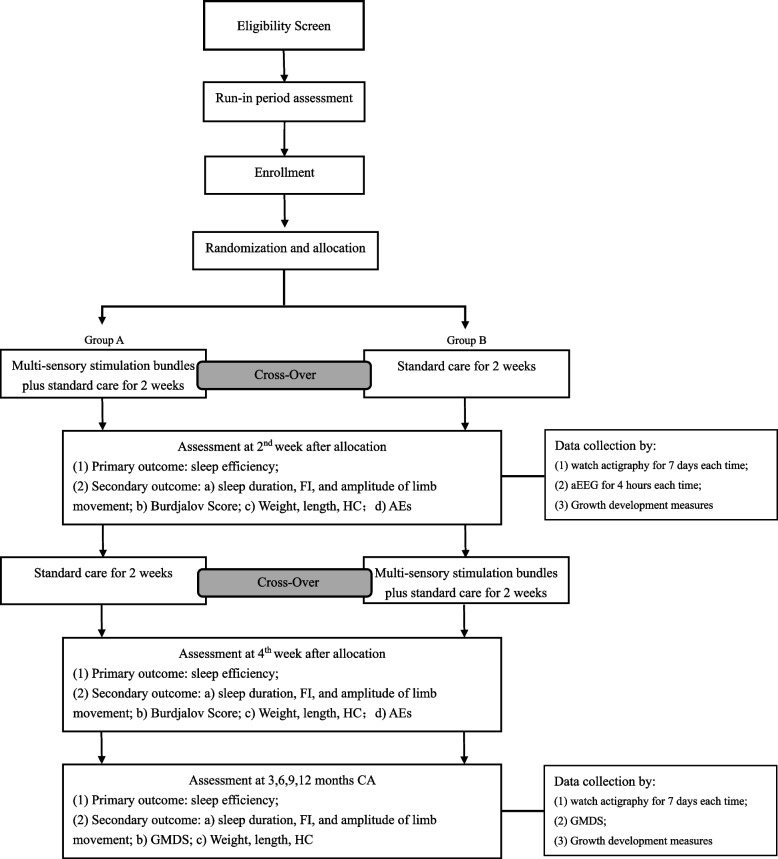
Patient flow

The screening will be performed by the study nurses, who will approach the eligible infants once they are stable. Owing to the fragile clinical status of VPIs, the study nurse and attending doctor will continuously monitor their clinical stability during the run-in period assessment. The criteria of clinical stability will be as follows: (1) when the vital signs of the infants are within the normal range according to the Neonatology book [27], including the temperature, heart rate, respiratory rate, and blood pressure; (2) when no vasoactive drugs or cardiopulmonary resuscitation are used for infants during the run-in period. Once the run-in period assessment is complete, the study nurse will open a sealed envelope containing the study identification number and group allocation and will inform the research team (see Patient flow, Fig. 1). Additionally, the study nurse will explain the experimental protocol, processes, and precautions to the parents of infants who meet the inclusion criteria. Finally, the study nurse will obtain signed letters of informed consent from the parents.

### Interventions with participants and investigation equipment

**Fig. 2:**
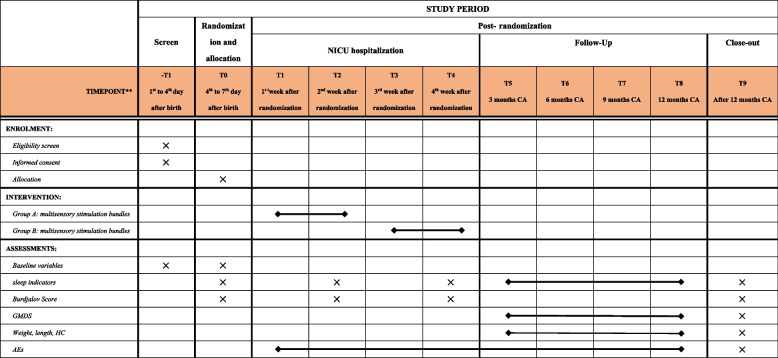
Schedule of enrolment, interventions, and assessments

Participants will receive two types of care—“standard care” and “standard care plus multisensory stimulation bundles”—randomly for two consecutive weeks each (see schedule of enrollment, interventions, and assessments in Fig. 2). Thus, every participant will receive the tested intervention. The difference between the groups will only be the order in which they receive the intervention. Furthermore, all healthcare providers will receive detailed training on the study process before the study commences. To ensure precise and standard sensory stimulations, we will provide the multisensory stimulation bundles using the multisensory stimulation-supported device. The device application’s standard operating procedure (SOP) has been developed. All healthcare providers involved in VPI care will be trained to apply the device and standard care through presentations and bedside demonstrations prior to the start of the study, ensuring intervention homogeneity.

### Standard care

The standard care will involve specific interventions, including reducing light intensity, noise level, posture support, and pain management [21], which will be implemented according to the SOP of NICU developmental care. Moreover, infants’ conditions will be assessed at a minimum of every hour or as needed by bedside nurses.

### Multisensory stimulation bundles

**Fig. 3:**
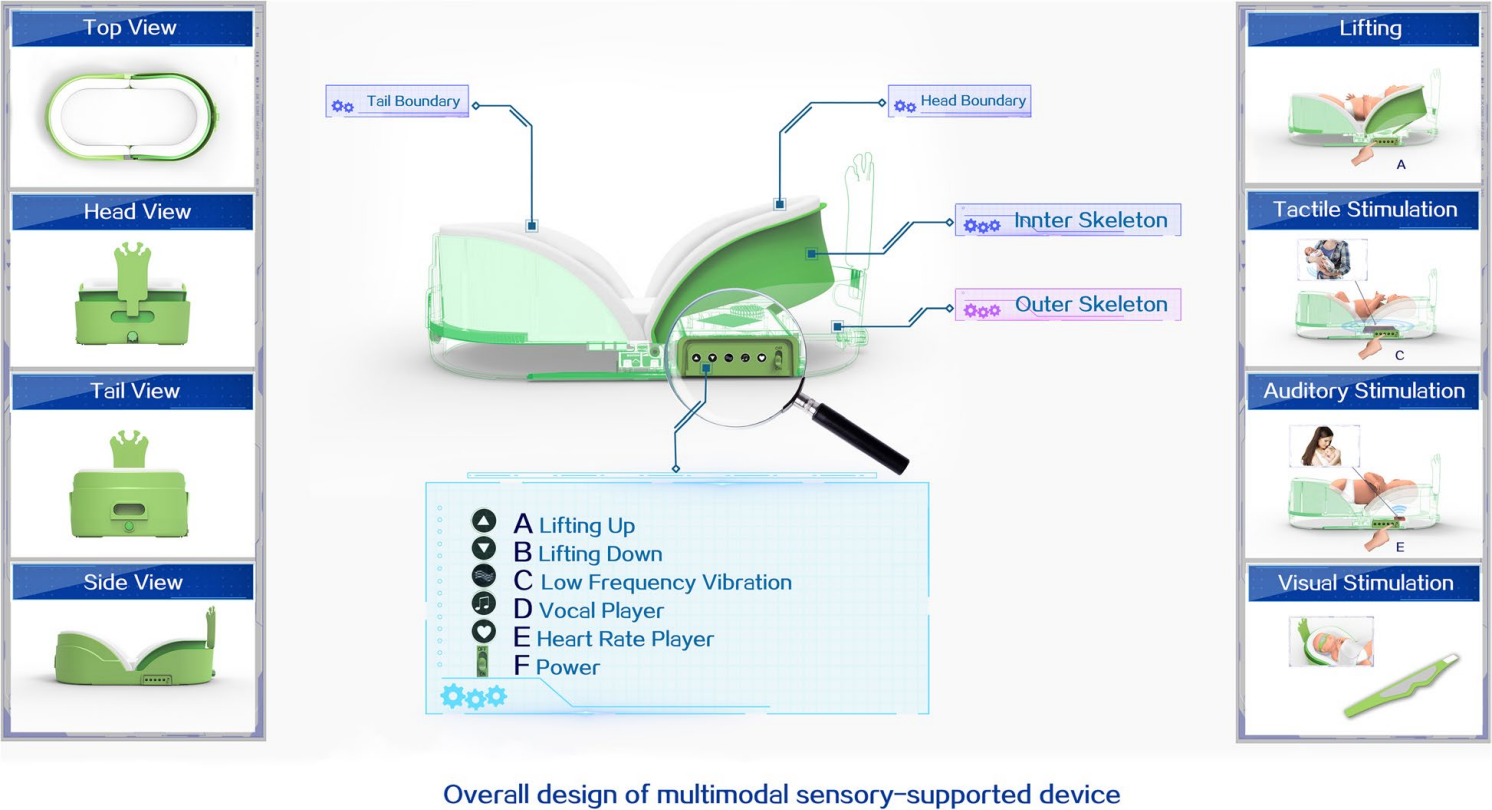
Overall design of device

**Fig. 4:**
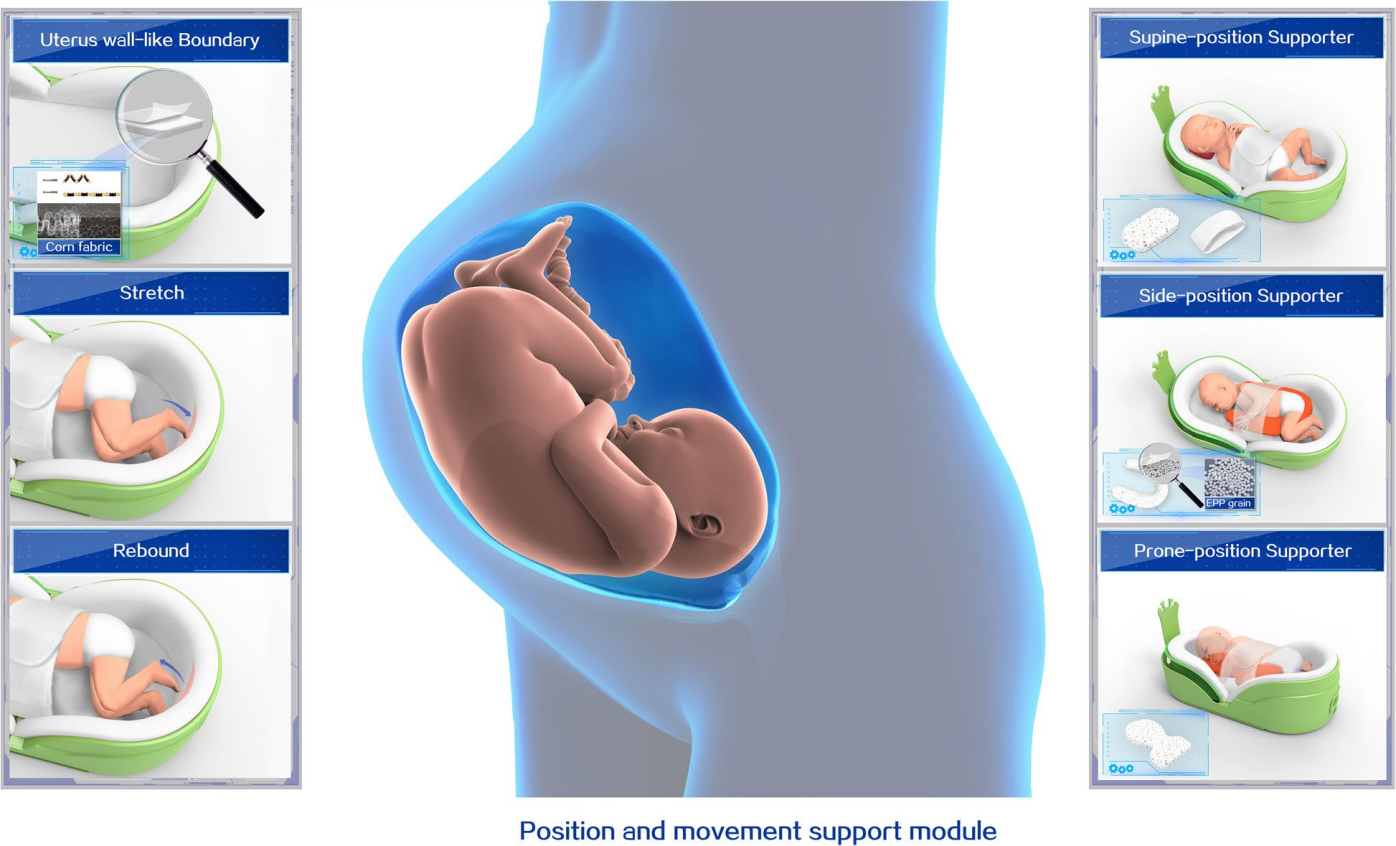
Position and movement support module

Based on the Enriched Environment recommendation, the multisensory stimulation bundles will be presented in this research [19]. Specifically, two stimuli mechanisms using the multisensory stimulation device will be implemented by trained staff. This device will be able to provide (i) multisensory stimulation bundles (i.e., vision, auditory, and tactile), which simulates maternal interaction with infants, and (ii) posture support similar to the intrauterine boundary-like enclosed physical space to allow the infant’s upper and lower extremities to touch the device wall and bounce back; this physical activity and sense of boundary experience will trigger a hand-to-mouth midline orientation movement, which could promote the neurobehavioral development of infants. The intervention bundles provided with the multisensory-supported device will be included as follows (see Figs. 3 and 4).

- Visual stimulation: Based on recent recommendations and evidence [18, 28], cycled light will be provided in a 12-h-on, 12-h-off pattern. Near-darkness (5–30 lux) will be provided using the designed eye mask except for procedures during night hours (7:00 PM to 7:00 AM). Meanwhile, daylight (200–600 lux) will be provided with folded incubator covers to allow light in from four sides during daytime hours (7:00 AM to 7:00 PM), which means the eye mask will be removed during this period. Moreover, the researcher will also assess lux measures for every infant weekly.
- Auditory stimulation: Based on the available evidence [15, 16], a vocal player will provide a recorded lullaby and maternal heartbeat for 15 min at 9:00, 12:00, and 19:00 after feeding (see Fig. 3).
- Tactile stimulation: A simulated tactile module has been developed based on the global massage protocol parameters [29, 30]. It aims to provide a sensation similar to parents’ gentle touch. The vibration ranges from the shoulders to the legs of the body and is produced outside the mattress. Low-frequency (18 Hz) vibration will be provided through tactile stimulation for 15 min twice a day (see Fig. 3). The frequency of vibrations is calculated according to the gesture strength of massage provided by parents or medical staff. The researcher accumulated massage strengths provided by 10 personnel (five parents and medical staff), converted them into vibration frequency, and identified the frequency intensity as 18Hz.
- A uterus-like boundary: This womb-simulating device allows infants to assume a similar position and movement as in the uterus. A corn-derived fiber was selected as the inner lining of the boundary. This fabric has a resilience force equal to the newborn’s maximum muscle strength to encourage their middle orientation and self-generated movements (see Fig. 4).

### Investigational equipment

Guided by global guidelines and current evidence, a multisensory-supported device was specifically designed to provide precise and standard multisensory stimulation bundles with smooth clinical adaptation for VPIs during hospitalization. The device is composed of inner and outer skeletons connected by articulated components. Several functional buttons are set on the outer skeleton, including vocal play and head–shoulder lift. More details on the device introduction can be found in Additional file 1.

Moreover, to ensure the safety of the “multisensory-supported device” equipment design, the researchers sent the study equipment to the Chinese National Medical Device Inspection Center for testing its safety. Preclinical studies began after receiving the product safety report. During the study, participants will be continuously monitored using an electrocardiography monitor and assessed by bedside nurses at 3-h intervals or as needed as part of routine care. The study nurse will check the fidelity of intervention delivery twice daily and record it in the checklist. Additionally, there will be at least one new device on standby to promptly replace any faulty devices for the infants. Moreover, the study nurse will check the parameters (i.e., lux measures) of the device in use weekly to ensure they stay within the predetermined range. The study checklist will also record the measurement outcome.

### Study outcomes

All infants will participate in the same assessment schedule regardless of group assignment. In this study, the period of crossover intervention will be no more than 5 weeks (including the run-in period assessment).

#### Primary outcome

The primary endpoint of this study is sleep efficiency, which is defined as the average proportion of sleep time for 7 days at weeks 2 and 4 (see Fig. 2). In this study, sleep is defined as three consecutive minutes with lower-extremity movement below the threshold. It is measured using an actigraphy watch (model: Actiwatch-2). Actigraphy is a reliable and valid method for continuously recording limb movements and transferring sleep parameters [31, 32]. Data from the Sleep Watch Actigraph will be coded into sleep and wake states at 1-min intervals using Action 4 research-level software (Ambulatory Monitoring, Inc, Ardsley, New York), compatible with the actigraphy watch. Sleep/wake measures will be extracted from actigraphy data by one investigator who is blind to the infants’ assigned groups. The actigraphy watch weighs 27 grams. Previous observations made at the study institution have shown that the device has minimal impact on infants’ activities. To further minimize its impact on the activities of preterm infants, the researcher will uniformly place the watch on the infant’s thigh.

#### Secondary outcomes

Secondary study outcomes will include the following variables:

- Other sleep indicators: Sleep duration, fragmentation index, and amplitude of limb movement will also be obtained using the actigraphy watch (model: Actiwatch-2).
- Burdjalov Score: The Burdjalov Score is a scoring system used to evaluate the brain maturation of neonates. It is measured through aEEG examinations (CFM3000). It comprises four component variables: (1) presence or absence of “sleep–wake cycling,” (2) continuity of the brainwave record pattern, (3) recording bandwidth, and (4) lower-border amplitude (in μV) (see Table 1). Each variable will be independently evaluated, and each participant’s scores will be summed to determine the total score for each recording [33]. The minimum and maximum possible total scores are 0 and 13, respectively. A qualified physician will conduct bedside aEEG examinations, which involve continuous data collection over 4 h at baseline, week 2, and week 4 after randomization (see Fig. 2). Afterward, the physician will interpret the aEEG data to calculate the Burdjalov Score. During the aEEG examination, the room temperature will be maintained at 24–28°C, while unnecessary iatrogenic procedures on the infant will be minimized.
- The Griffiths Mental Development Scales (GMDS) with six subtests will be used to assess neurodevelopment in children aged 0–2 years [34, 35]. Each subtest measures a different development area: (A) locomotor, (B) personal–social, (C) hearing and language, (D) eye and hand coordination, (E) performance, and (F) practical reasoning. The mental age (MA) is derived by calculating the average raw scores of the six subtests. The corrected age (CA) is calculated as the difference between the examination and birth dates. The developmental quotient (DQ) is expressed as a mental ratio, i.e., DQ = (MA / CA) × 100. Finally, the total development quotient is represented by GQ, which represents the average value of all the development quotients [35]. The scale is culturally appropriate for Chinese children [35]. In this study, two physician research assistants with international GMDS qualifications will conduct GMDS assessments on study participants. Furthermore, the assessment process will be video-recorded for possible replay. Interrater measurement consistency will be obtained prior to the actual assessment of infants to reduce errors.
- Growth indicators at follow-up will include weight, length, and head circumference. Moreover, the research assistant will collect data when participants return for follow-up at 3, 6, and 12 months of CA.

**Table 1:** The Burdjalov Score

Score	Continuity	Sleep-wake cycling	Amplitude of lower border	Bandwidth span and amplitude of lower border
0	Discontinuous	None	Severely depressed (<3 µV)	Very depressed: low span (≤15 µV) and low voltage (5 µV)
1	Somewhat continuous	Waves first appear	Somewhat depressed (3–5 µV)	Very immature: high span (>20 µV) or moderate span (15–20 µV) and low voltage (≤5 µV)
2	Continuous	Not definite, somewhat cycling	Elevated (>5 µV)	Immature: high span (>20 µV) and high voltage (>5 µV)
3		Definite cycling, but interrupted		Maturing: moderate span (15–20 µV) and high voltage (>5 µV)
4		Definite cycling, noninterrupted		Mature: low span (<15 µV) and high voltage (>5 µV)
5		Regular and mature cycling		

### Adverse events (AEs)

In this study, AEs will refer to all adverse medical events that occur during the study period, which may be manifested by signs, symptoms, disease, or abnormal laboratory tests. According to Common Terminology Criteria for Adverse Events (CTCAE), AEs in this study include impaired skin integrity, skin allergies, fever, and fluctuating oxygen saturation, even the disease deterioration requiring surgery, cardiopulmonary resuscitation, and ventilator parameter model upgrade, among others. The CTCAE, a grading scale helps to determine and assess the severity of a clinically adverse event, which displays grades 1 through 5 (indicating mild, moderate, severe, life-threatening consequences, and death) [36]. Meanwhile, WHO-UMC (UMC, Uppsala Monitoring Center) displays the association of AEs with experimental interventions (indicating certain, likely, possible, unlikely, conditional/unclassified, nonassessable/unclassifiable) [37]. The study will be terminated, when any AEs that are likely or certain to be associated with intervention or severe AEs that are possibly, likely, or certain related to the intervention. The AEs will be recorded in a case report form (CRF) within 24 h of occurrence. Severe AEs will be immediately reported to the Hospital Research Ethics Committee, followed by suspension of the study. The AE occurrence ratio will be calculated and analyzed.

### Control variables and covariates

To characterize the sample and ensure balance across groups in analyses for treatment effects, we will prospectively collect the following information from electronic medical records and parent report questionnaires to control for the following: (1) major medical complications associated with prematurity, including GA, birth weight, sex, history of antenatal steroid usage, infection, number of days of intubation and oxygen, and X-ray changes consistent with chronic lung disease; (2) postmenstrual age at sleep and aEEG measurement; and (3) the number of days of exposure to study treatment. We will also obtain metrics to ensure balance across groups. If the groups do not match, we will use the unmatched metric as a covariate in the analyses.

### Data collection

All data collection collaborators will receive detailed training on the study process before starting the study to ensure the homogeneity and integrity of data collection. During the hospital stay, outcome measurements will be conducted by reviewing medical records, including medical notes and nursing care charts, as well as using the Actiwatch-2 Actigraph, aEEG, and GMDS for data collection. Further, The data collectors must have obtained the necessary licenses, such as aEEG and GMDS qualifications. Data collected after withdrawal of participation and the participants’ hospital mortality would be the only reasons for the non-inclusion of data. All forms completed by the outcome measurement, screening, and enrollment teams will be entered into an electronic platform; and two staff members will work together on data entry to minimize input mistakes. The Epi Data software will be used to control the range and accuracy of the data entered. The schedule for outcome assessments is shown in Fig. 2.

### Data quality and monitoring

For this study, an eCRF record must be completed for each patient. Data will be anonymized by assigning a unique code for each participant. The critical document containing each patient’s name related to the code number will be stored in the folder of the principal investigator (PI) at each center. Data will be kept in an institutional research location of the PI, secured with a password or key for the period specified by legislation. The SCMC Clinical Research Department will engage relevant experts to form an independent data monitoring committee (IDMC). The IDMC will conduct periodic monitoring visits during the trial, which will include data collection, patient safety, and research progress.

### Sample size calculation

In the previous pilot study conducted during October 2021 to February 2022, five participants in the intervention group vs five with matched characteristics in the control group were compared. The primary outcome (defined as sleep efficiency) was found to be 0.52 and 0.48 in the intervention and control group, respectively. The intervention effect size was estimated to be 0.04, and the inter-person standard deviation, 0.05. Considering a two-tailed significance level of 5% (α) and a test power of 90%, the required sample size is 36, with 18 in each group. Considering a dropout rate of 10%, 40 participants will be recruited for this study. In the past 5 years, the study setting received more than 100 VPIs every year, which means the sample size could be achieved in the planned time frame.

### Data analysis

The baseline characteristics of the samples will be presented as *N* (%) for categorical variables and mean ± SD or median (IQR) for quantitative variables, depending on the data distribution. The primary and secondary outcomes will be analyzed using a linear mixed-effect model with “intervention” as a between-subjects factor and “time” as a within-subjects factor. The primary outcome (sleep efficiency) will be evaluated at timing points of week 2 (T2 timing point) and week 4 (T4 timing point) after randomization during the hospitalization. We compared the mean difference between primary and secondary outcomes as follows: group A (T2) vs. group B (T4) and group A (T4) vs. group B (T2). Moreover, every participant will receive the investigated intervention, and the difference between the groups will be the order in which they receive it. Therefore, no statistical difference would exist between groups during the follow-up period. Hence, we will only conduct the statistical description of the follow-up indicators. No statistical test will be prespecified regarding the follow-up indicators in this study. The intention-to-treat analysis will be used for missing values during each data collection. Although cases of missing data are expected to be rare, multiple imputations assuming random missing data will be used for this study. SPSS software version 24.0 (IBM Corp., Armonk, NY, USA) will be used for data analysis. Two-tailed *p*-values < 0.05 will be considered indicative of statistical significance.

## Discussion

In the last decade, research on sensory-based interventions has been highly emphasized. It has been requested that the procedure and impacts of the interventions show precise steps and be objectively measured during NICU hospitalization, particularly for very and extremely preterm infants [9, 25]. However, clinical staff or parents provide most primary sensory stimulation for infants in a busy neonatal care unit. Therefore, ensuring the sensory stimulation is provided with standard procedures, precise details, and proper doses of stimuli throughout NICU hospitalization [9, 23, 24] may be challenging. Directing this research toward addressing the clinical utilization gap is an urgent matter that needs to be resolved to advance clinical practice for highly preterm infants and improve their sleep and neurobehavioral outcomes.

The intervention protocol of this study differs from traditional interventions as it creates an infant care device that could produce a precise and consistent stimulation similar to the maternal tactile, auditory, posture, and visual effects for hospitalized preterm infants and VPIs. This study presents the first multisensory stimulation device that integrates a multimodal care bundle based on global guidelines and solid clinical evidence. Furthermore, simultaneously, this study will provide new perspectives on strategies for parent and infant reciprocal interaction that are critical for sleep and neurobehavioral development, particularly for NICUs that must limit the frequency of visits because of the COVID-19 pandemic.

The hypothesis of this study protocol is that preterm infants who receive the multisensory stimulation bundle protocol using the multisensory stimulation device will have improved sleep, early neurodevelopment outcomes, and growth development within the early period of infancy. Moreover, the study investigators will observe the safety, clinical feasibility, and applicability of this newly designed multisensory stimulation device. If the hypothesis is confirmed, the results could further generalize the implementation of protocols of multisensory stimulation bundles in NICUs and indicate that the device is an effective and low-cost measure to facilitate sleep and early neurodevelopment of hospitalized preterm neonates.

### Limitations

Although this device can provide a womb-like surrounding environment, promoting infants’ flexion and midline orientation activities, the surrounding textile boundaries could lead to slow heat dissipation. However, the nurses can control the infants’ temperature by monitoring and adjusting the temperature and humidity of the incubator. Thus, a design regarding heat-loss material should be further investigated. Another limitation of this study might be that the actigraphy watch has not been validated for the measurement of sleep in neonates. More studies regarding the validation of actigraphy watch against polysomnography among a large sample of preterm population are needed.

## Conclusion

This study will be the first that provides multisensory intervention bundles through an integrative device for VPIs in NICU settings. It will identify the effects of a multisensory stimulation bundle protocol on VPIs in terms of sleep, early neurodevelopmental outcomes, and physical growth developmental outcomes. Furthermore, the researchers in this study will demonstrate the safety, acceptability, and feasibility of implementing multisensory intervention bundles in NICU settings to benefit infants, health caregivers, and parents. This study is expected to have a clinical impact on very preterm care during hospitalization and over 1 year of life post-NICU discharge.

## Trial status

The study commenced recruiting in April 2022 with a projected completion in December 2023. Recruitment is live at Shanghai Children’s Medical Center, affiliated with Shanghai Jiaotong University School of Medicine.

### Supplementary Information

**Additional file 1. **Multisensory-supported device introduction.

**Additional file 2.**

## Data Availability

De-identified data analyzed in the current study will be made available upon reasonable request from the corresponding author.

## Abbreviations

aEEG: Amplitude-integrated electroencephalography
AE: Adverse event
AS: Active sleep
ASE: Active sleep efficiency
CA: Corrected age
CRF: Case report form
CTCAE: Common Terminology Criteria for Adverse Events
DQ: Developmental quotient
GA: Gestational age
GMDS: Griffiths Mental Development Scales
IS: Indeterminate sleep
MA: Mental age
NICU: Neonatal intensive care units
PI: Principal investigator
QS: Quiet sleep
REM: Rapid eye movement
SOP: Standard operating procedure
TST: Total sleep time efficiency
VPI: Very preterm infants
